# The summer indoor temperatures of the English housing stock: Exploring the influence of dwelling and household characteristics

**DOI:** 10.1177/0143624419847621

**Published:** 2019-05-06

**Authors:** Giorgos Petrou, Phil Symonds, Anna Mavrogianni, Anastasia Mylona, Mike Davies

**Affiliations:** 1 Energy Institute, University College London, London, UK; 2 Institute for Environmental Design and Engineering, University College London, London, UK; 3 CIBSE, London, UK

**Keywords:** Overheating, thermal comfort, indoor temperature, England, dwellings, households

## Abstract

As the high temperatures experienced during the summer of 2018 may become commonplace by 2050, adaptation to higher indoor temperatures while minimising the need for mechanical cooling is required. A thorough understanding of the factors that influence indoor temperatures can enable the design of healthier and safer dwellings under a warming climate. The aim of this paper is to provide further insight into the topic of indoor overheating through the analysis of the largest recent sample of English dwellings, the 2011 Energy Follow-Up Survey, comprised of 823 dwellings. Following the pre-processing stage, the indoor overheating risk of 795 living rooms and 799 bedrooms was quantified using the criteria defined within CIBSE's Technical Memorandum 59. Approximately 2.5% of the dwellings were found to exceed Criterion 1, with this figure approaching 26% when Criterion 2 was considered. Subsequently, the indoor temperatures were standardised against external weather conditions and the correlation of 11 dwelling and 9 household characteristics was examined. Factors such as the main heating system, tenure and occupant vulnerability were all found to have a statistically significant association with the indoor temperatures. Further analysis revealed multiple correlations between household and dwelling characteristics, highlighting the complexity of the indoor overheating problem.

***Practical application***: By applying the criteria in CIBSE's TM59, 26% of the dwellings monitored during the 2011 Energy Follow-Up Survey were found to overheat. Since 2011 was a cool summer and future temperatures are expected to be warmer, even more dwellings are expected to fail these criteria in the future. Multiple dwelling and household characteristics were associated with higher indoor temperatures, including: dwellings with a SAP rating > 70, more recently built and with communal heating. Thus, it is crucial to consider indoor overheating risk at the building design or refurbishment stage to prevent the possible consequences of uncomfortably high indoor temperatures.

## Introduction

The summer of 2018 was the hottest on record in England.^1^ As a result of anthropogenic greenhouse gas (GHG) emissions,^2^ the chance of such high summer temperatures will increase from less than 10% (1981–2000) to approximately 50% by 2050.^3^ To mitigate the worst of the possible consequences of climate change, the UK introduced in 2008 the Climate Change Act and set the aim of reducing GHG emissions to 80% below the 1990s levels by 2050.^4^ With increased levels of building thermal insulation and airtightness, along with the use of better-performing boilers, the GHG emissions of the domestic stock were reduced by 22% compared to 1990s levels, despite a 25% increase in the number of homes.^5^ Alongside further reductions in the GHG emissions of homes,^5^ the need for adaptation to the higher indoor temperatures associated with the increased ambient temperatures is also clear,^6,7^ especially since people spend most of their time indoors.^8^ Higher outdoor temperatures can drive indoor thermal discomfort, a phenomenon referred to as indoor overheating,^9^ which could impair the occupants sleep^10,11^ and general wellbeing.^6^ During periods of extreme hot weather, the increased heat stress could also result in serious health consequences and even death, especially for older people or individuals with illnesses and physical disabilities.^12^ During the 10-day period of the 2003 heatwave, the mortality rate of people aged 75 or older by location of death increased in Southern England by: 33% in their own home, 42% in nursing homes and approximately 29% in residential homes^13^ (although the harvesting effect may have influenced the exact figures).^14^

Although the possible implications of focusing on winter thermal comfort instead of whole-year thermal comfort have long being debated,^15^ presently, an assessment of indoor overheating risk is not enforced within the building regulations.^16^ The Approved Document L1A for new dwellings suggests the use of a steady-state model described in Appendix P of SAP 2012 to ensure that summer heat gains are limited^17^ while the equivalent document for existing dwellings, Approved Document L1B, does not provide any advice on the assessment of indoor overheating.^18^ Numerous concerns regarding the efficacy of the SAP 2012 model in identifying dwellings at high risk of indoor overheating have been raised.^16^ In the recent release of SAP 10, which is not currently used for official purposes, the indoor overheating assessment was improved through the option for reduced air-change rate in the case of noise or security concerns.^19^ However, the same steady-state model and monthly mean temperatures as in SAP 2012 are used, with no distinction between bedrooms and living rooms being made. Thus, concerns regarding the ability of this method to adequately capture the stochastic nature of human behaviour or account for future heatwaves may be raised. Nonetheless, evidence from the application of the SAP 10 model are required before drawing any final conclusions. An alternative approach for the assessment of indoor overheating risk at the building design stage was provided by the Chartered Institution of Building Services Engineers (CIBSE) with the release of Technical Memorandum 59 (TM59).^20^ The method is based on the use of building performance simulation (BPS) tools, with two threshold criteria defined based on the theory of adaptive thermal comfort and previous research on sleep quality.^21–23^ However, these criteria have been challenged, since they are based on old field studies primarily in offices that may not capture adequately the occupants' adaptive capacity.^24,25^ Therefore, despite the plethora of modelling and monitoring studies on the drivers of indoor overheating risk,^26–30^ our understanding of how to effectively quantify and reduce indoor overheating risk at the building design or refurbishment stage is incomplete.

This study aims at providing further evidence on how dwelling and household characteristics may influence indoor temperatures. This will be achieved through the statistical analysis of the largest recent monitoring campaign of English dwellings, the 2011 Energy Follow-Up Survey (EFUS) with a sample of 823 dwellings. Specifically, this study seeks to answer the following questions:

1. What is the indoor overheating risk of the monitored EFUS dwellings according to the threshold criteria defined in TM59?
2. Which dwelling and household characteristics have a statistically significant association with higher indoor temperature?
3. Which dwelling and household characteristics are statistically correlated?

This will be the first England-wide assessment of indoor overheating risk using the TM59 criteria, while the statistical investigation of the factors that may influence the summer indoor temperatures aim to inform our adaptation efforts to a warming climate.

## Literature review

Over the last few years, there have been a series of monitoring campaigns that investigated the factors that influence summer indoor temperatures in the UK. A comprehensive summary of recent monitoring campaigns was provided by Pathan et al.^26^ and Fosas et al.^31^ with some key findings discussed below.

Within a monitoring campaign of 55 dwellings located in Exeter, vulnerable households (with older occupants or occupants with illnesses and physical disabilities) and overcrowded households were exposed to higher mean temperatures than non-vulnerable and non-overcrowded households.^27^ In a 2009 monitoring campaign that involved 230 dwellings in Leicester, occupants aged over 70 years were more likely to heat their homes over the summer.^30^ The same study also revealed that heated homes were amongst the 13% warmest homes monitored and that typology is another influential factor with flats identified as the warmest. A London monitoring campaign of 122 dwellings over the summers of 2009 and 2010, identified overheating as a widespread problem with 75% of the bedrooms failing at least one of the two fixed overheating thresholds used.^26^ From this result, the authors concluded that targeting particular categories of dwellings may not adequately address the issue of indoor overheating.^26^ The monitoring of eight social housing London dwellings, indicated that indoor overheating is already experienced even during mild summers, although the severity depends on the criterion used.^28^ A post-occupancy evaluation undertaken in 26 Scottish dwellings built after 2009 demonstrated that indoor overheating risk is not localised to the southern United Kingdom.^32^ A few dwellings were found to overheat even during non-summer periods and numerous dwelling characteristics (e.g. heat loss parameter) and occupant-related actions (e.g. thermostatic control) were identified as influential. Another key point raised was the discrepancy between indoor overheating assessments and stated thermal discomfort – occupants of some dwellings with a relatively high percentage of overheating hours did not identify overheating as a problem, with the opposite being true for a few dwellings with a relatively low number of overheating hours recorded.^32^

A common limitation between the monitoring studies in English dwellings discussed above is the focus on a single location. An exception is the 2007–2008 nationwide Carbon Reduction in Buildings (CaRB) study of 207 dwellings.^33^ The static overheating criteria and mean monitored temperatures over the summer period were used to determine whether significant differences exist due to location, external wall type, age band and building type. However, as suggested by the literature,^27,32,33^ numerous other factors could influence the indoor environment. In addition, as the distribution of characteristics (e.g. building type) is not necessarily uniform between different regions, a statistical comparison of mean temperatures or static thresholds is likely influenced by the local weather.

During the 2010/2011 EFUS, 2616 households were interviewed, with 943 receiving temperature loggers and 823 returning them with adequate data for analysis.^34^ Overall, 20% of the interviewees reported difficulty in keeping at least one room cool during the summer months and identified insufficient shade as the primary reason for overheating. Bivariate analysis on the factors that influence the likelihood of occupants expressing thermal discomfort suggested dwelling age, floor area and location to all be important. Households with SAP rating > 70 were associated with a higher thermal discomfort and so were occupants of registered social landlord (RSL) dwellings. Larger households or households without a pensioner present were also more likely to report thermal discomfort. Despite the thorough analysis of stated thermal discomfort, the analysis conducted on the summer indoor temperatures was limited. Hulme et al. did not differentiate between bedrooms and living rooms which could be an important distinction due to their different use. In addition, as the prediction of indoor overheating risk is based on indoor temperatures and the association between temperatures and thermal discomfort is yet unclear, a detailed analysis of indoor temperatures would complement the analysis of Hulme et al.

## Methods

The English Housing Survey (EHS) is a national survey, commissioned by the Ministry of Housing Communities and Local Government (previously Department for Communities and Local Government), that takes place every two years and consists of household interviews and physical surveys.^35^ The interviews typically cover topics such as demography, employment and income while the surveys gather information regarding the dwelling conditions and energy efficiency measures.^36^ As a follow up to the 2010–2011 EHS, the EFUS survey conducted further interviews and surveys in 2616 dwellings with the purpose of updating the modelling assumptions regarding how energy is used at home.^36^ For 943 dwellings, the indoor air temperature was monitored using TinyTag Transit 2 loggers^36^ at 20-minute intervals in the living room, bedroom and hallway from the time of installation (December 2010 to April 2011) until they were returned (April/May 2012). Adequate data for at least one room were returned by 823 dwellings. The monitored temperatures, interviews and survey data can be linked to the data within the EHS through access to the UK Data Service.^37–39^

**Figure 1.**
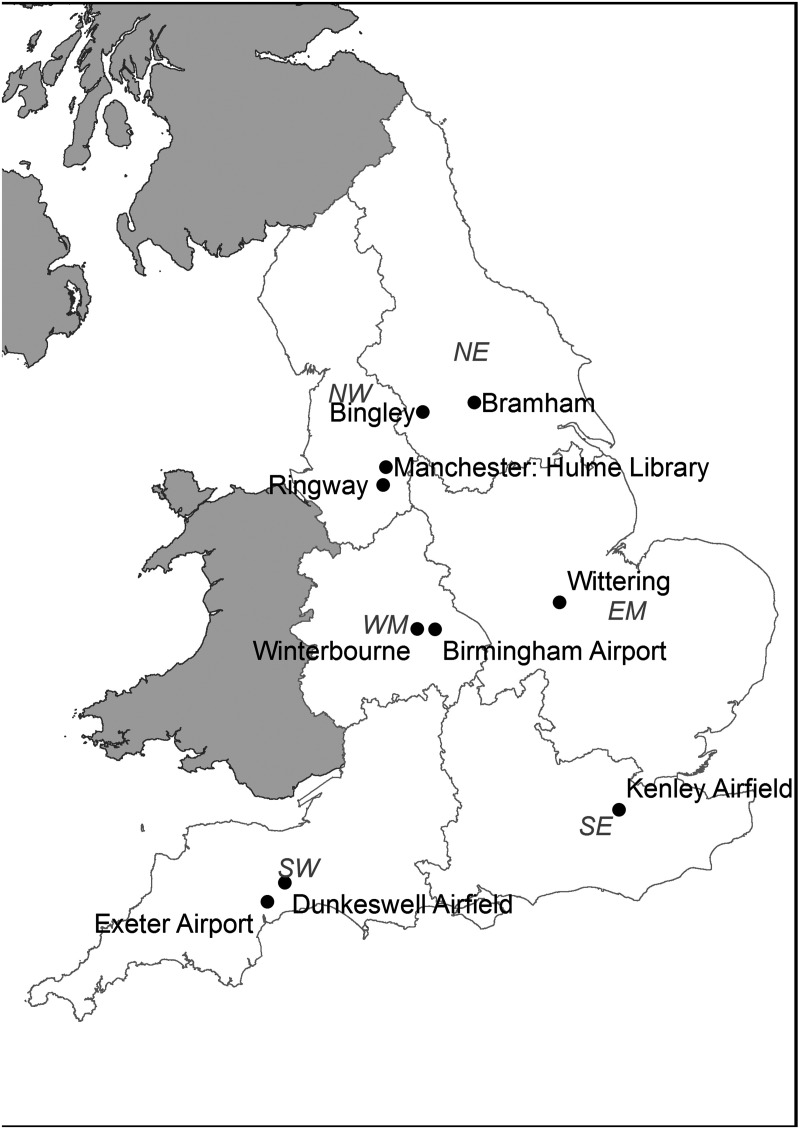
Location of the weather stations used for each region. Reproduced from Symonds et al.^41^

**Figure 2.**
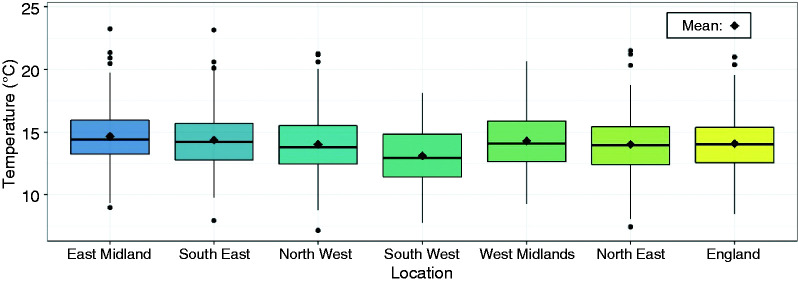
Box plots of the daily mean temperature recorded by the six weather stations between May and September. The box-plot of England represents the average daily mean temperature across the stations.

Weather data were obtained from the Met Office Integrated Data Archive System (MIDAS) database^40^ for the weather stations in the six regions identified in Figure 1 and as described in more detail by Symonds et al.^41^ A summary of the daily-mean temperature of each region is provided in Figure 2. Depending on its Government Office Region (GOR), each dwelling was associated with one of the six regions. At the pre-processing stage, the monitored indoor temperatures of each dwelling were analysed with the purpose of identifying extreme values that could be the result of faulty or misplaced data loggers (e.g. positioned near heat sources). Given the relatively cool conditions during the summer of 2011, individual recordings that exceeded 40℃ were removed and the temperatures measured at 20-minute intervals were averaged to give hourly values. In the case that multiple recordings exceeded 40℃, that logger was removed from the dataset. Subsequently, for each region, the temperature profiles of statistical outliers were qualitatively assessed to determine whether further elimination was required (e.g. in case of year-long flat temperature profiles). In the case of missing data from bedroom or living room loggers during the period May–September (inclusive), the rooms of these dwellings were not included in the overheating assessment. Following the pre-processing stage and from an initial sample of 823 dwellings, the temperatures monitored in 795 living rooms and 799 bedrooms were considered adequate for analysis.

### Indoor overheating assessment

To translate indoor temperatures into overheating risk, the two criteria defined in TM59^20^ for naturally ventilated dwellings were used. This does not imply that this is a validation study of TM59, a design stage guidance that is based on the use of BPS tools. However, as TM59 is the only overheating guidance focused on dwellings, it was deemed appropriate to utilise the same criteria. A form of these criteria, but not the combination of, has been used in the past to assess overheating risk in previous in-use studies.^27,30,42^ A high risk of indoor overheating was assumed if either of the following thresholds is exceeded:^20^

1. The percentage of occupied hours where the operative temperature (*T*_*op*_) exceeds the maximum allowable temperature (*T*_*max*_) by 1℃ or more during the period May to September, inclusive, should not exceed 3%.
2. Bedroom operative temperature should not exceed 26℃ for more than 1% of the assumed sleeping hours (22:00–07:00) annually (equivalent to 32 h).

Local weather data were used to estimate a *T_max_* for each region using the equations in CIBSE TM52.^9^ The dwellings were assumed to be predominantly naturally ventilated, with the living room being occupied between 09:00 and 22:00 and the bedroom being always occupied, as suggested in TM59.^20^ As only the air temperature was monitored, this was used instead of the operative temperature in the overheating risk assessment. This assumption does not capture the influence of radiant temperatures on thermal comfort.^9^ However, given the data available, this limitation could not be overcome.

### Standardisation of indoor temperatures

To account for the inter-regional variations in local weather, the indoor temperatures were standardised against the external conditions to allow for a direct comparison between the monitored temperatures of the entire housing stock. A similar method has been used in the past to analyse winter temperatures^43,44^ and enables the nationwide investigation of factors that influence the indoor environment.

Amongst the numerous models evaluated, a balance was struck between model efficacy and simplicity for a model that was based on linear terms of daily-mean outdoor temperature ($T_{\mathrm{out},\mathrm{mean}}$) and Global Horizontal Irradiance (*GHI_mean_*) as described by the following equation

(1) $$\mathrm{SI}T_{\mathrm{room}}=\alpha_{0}+a_{1}T_{\mathrm{out},\mathrm{mean}}+a_{2}\mathrm{GH}I_{\mathrm{mean}}$$

*SIT_room_* is the mean day-time (08:00–22:00) indoor temperature estimated for the living room or the mean night-time (22:00–08-00) temperature for the bedroom. The models were trained only for the period of May to September (inclusive) due to the study's focus on the summer conditions. As it is good practise for a model's inputs to not differ significantly from the training data range, a daily-mean temperature of 20℃ was selected for the standardisation. This value was at the upper limit of the training dataset as shown in Figure 2. The mean value of *GHI_mean_* value of 210 W was the average GHI across the days that the outdoor temperature exceeded 19℃.

### Statistical analysis

The Kruskal–Wallis test was used to assess whether statistically significant differences exist for the SIT of the household and dwelling variables summarised in Tables 1 and 2. This test has been used previously for a similar analysis,^43^ it does not assume normality and it is able to deal with extreme data. The null hypothesis was that for each explanatory variable (that satisfied all assumptions):

**Table 1.** Summary of the household variables analysed.

Variable names	Value
Household composition	Couple, no dept. child(ren) < 60; couple, no dept. child(ren) ≥ 60; couple with dept. child(ren); lone parent with dept. child(ren); other multi-person households; one person < 60; one person aged 60 or over
No. of persons in the household	1; 2; 3; 4; 5; 6 or higher
Age band of youngest person	0–4; 5–10; 11–15; 16–24; 25–59; 60–74; 75–84; 85 or more
Age band of oldest person	16–34; 35–49; 50–59; 60–74; 75–84; 85 or more
Employment status of HRP and partner	One or more work full time; one or more work part time; none working, one or more retired; none working, and none retired
Extended tenure of household	Own with mortgage; own outright; privately rent; rent from LA; rent from RSL
Anyone illness or disability	Yes; No
All households - income in 5 bands	Lowest 20%; quintile 2; quintile 3; quintile 4; highest 20%
Household vulnerable – on means tested or certain disability related benefits	Yes; No

**Table 2.** Summary of the dwelling variables analysed.

Variable names	Value
Dwelling type	Bungalow; converted flat; detached; end terrace; mid terrace; purpose built flat; semi detached
Dwelling age	Pre 1850; 1850–1899; 1900–1918; 1919–1944; 1945–1964; 1965–1974; 1975–1980; 1981–1990; post 1990
Floor area	Less than 50 m^2^; 50 to 69 m^2^; 70 to 89 m^2^; 90 to 109 m^2^; 110 m^2^ or more
Storey	1st; 2nd; 3rd; 4th; 5th or higher
Construction	Solid masonry; cavity masonry; timber frame; steel frame; concrete frame; concrete boxwall
Double glazing	No double glazing; less than half; more than half; entire house
Nature of area	City centre; other urban centre; suburban residential; rural residential; village centre
Traffic problems	Yes; No
Main heating system	Boiler system with radiators; storage radiators; room heater; communal
Loft insulation	none; less than 100 mm; 100 up to 150 mm; 150 mm or more
SAP 09	Less than 30; 30 to 50; 51 to 70; more than 70

*The median SIT across the different levels (sub-groups) of each explanatory variable is the same at a signficance level of 5%*.

The *p*-value is a measure of how likely the observed data are under the null hypothesis, between 0 for impossible and 1 for certain.^45^ The researcher must decide in advance what p-value is the maximum acceptable (significance level); this is often set at 0.05. If the observed *p*-value is less or equal to 0.05, the data are unlikely under the null hypothesis and this gives evidence that a genuine difference exists. Therefore, if the *p* ≤ 0.05 for any variable (e.g. dwelling type), there is enough evidence to support a statistically significant difference between the median SIT of the variable's levels (e.g. bungalow, detached, etc.). If the variance of each level differed sligthly, stochastic dominance could still be demonstrated and this is indicated by an asterisk next to the *p*-value.^46^ This suggests that the SIT are still significantly different but it is not necessarily true that their median values are different. For each level, the pairwise Mann–Whitney *U*-tests for multiple comparisons with the false discovery rate (FDR) p-adjustment method was also performed. A *p*-value smaller or equal to 0.05 indicates a statistically significant difference between that level's SIT and that of the first level of each variable. To determine whether the dwelling characteristics are correlated to the household characteristics, Fisher's exact test was used with the null hypothesis of:

*There is no statistical association between categorical explanatory variables at a signficance level of 5%*.

If the *p*-value for any combination of variables (e.g. household composition and tenure) was less or equal to 0.05, a statistically significant association was assumed. For the above analysis, cases where the occupants did not provide an answer or stated that the survey question is not applicable to them were excluded.

## Results

**Figure 3.**
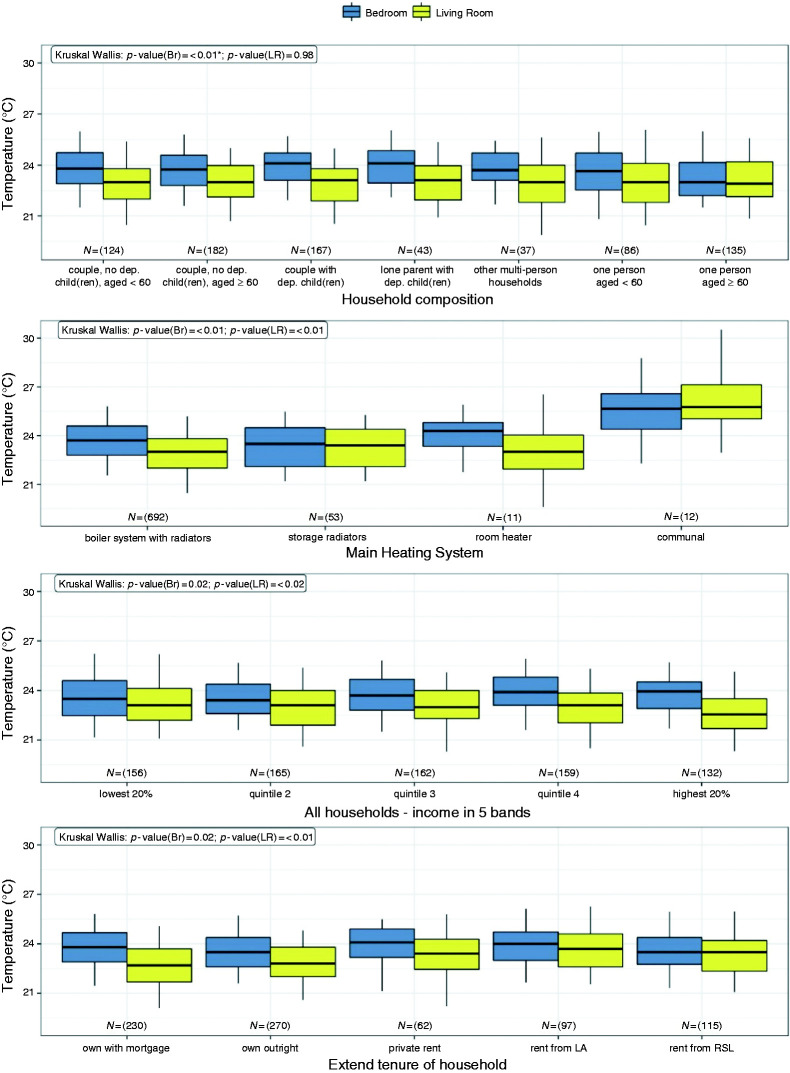
Box plots of standardised indoor bedroom and living room temperatures. The whiskers represent the 5^th^ and 95^th^ percentile. Outliers were masked for data privacy reasons. * on *p*-values indicates groups where the assumption of equal variance was not met but where the stochastic dominance could be assessed.

**Figure 4.**
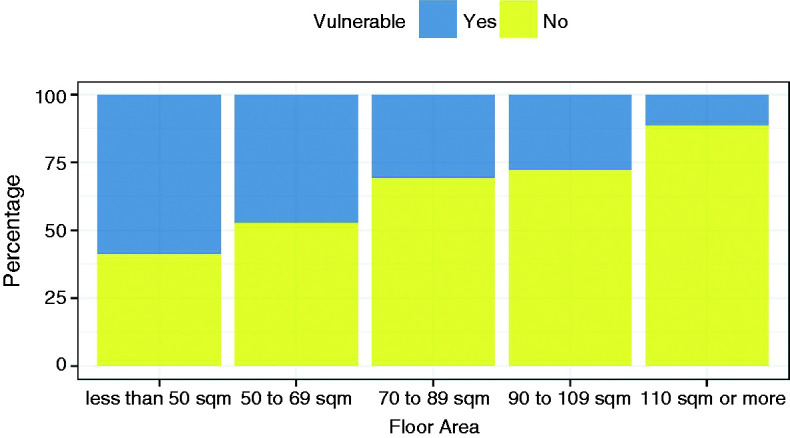
Bar plots of association between floor area and household vulnerability (defined in EFUS as dwellings on means tested or certain disability benefits).

Following the methods discussed above, the indoor overheating risk was estimated for 795 living rooms and 799 bedrooms, with the results summarised in Table 3. Subsequently, the relation between the SIT and the dwelling and household characteristics was explored, with the results summarised in Tables 4 to 7 and visualised in Figure 3. Finally, the correlation between dwelling and household characteristics is shown in Table 8 and Figure 4.

**Table 3.** Summary of the TM59 assessment results for the bedroom (B) and living room (LR) of each dwelling.

		Number of dwellings with percentage of overheating hours (%OH) by range		
Criterion	Total sample size	0	0 < %OH ≤ 3	%OH > 3
Criterion 1: LR	795	597	178	20
Criterion 1: B	799	496	284	19
		0	0 < %OH ≤ 1	%OH > 1
Criterion 2	799	377	218	2 4

**Table 4.** Summary of the median standardised indoor temperatures (SIT), 95% confidence interval (CI) and significance test results. The *p*-values associated with each variable are the results of the Kruskal–Wallis test and the *p*-values associated with each level of the variable are the result of the pairwise Mann–Whitney *U*-tests.

		Bedroom SIT (℃)		Living room SIT (℃)	
	*N*	Median (CI 95%)	*p*-Values	Median (CI 95%)	*p*-Values
Household composition	–	–	**< 0.01** *	–	0.98
Couple, no dept. child(ren) < 60	124	23.8 (23.5, 24.2)	–	23 (22.6, 23.3)	–
Couple, no dept. child(ren) ≥ 60	182	23.8 (23.5, 24)	0.76	23 (22.7, 23.3)	0.99
Couple with dept. child(ren)	167	24.1 (23.8, 24.2)	0.48	23.1 (22.8, 23.4)	0.99
Lone parent with dept. child(ren)	43	24.1 (23.3, 24.5)	0.83	23.1 (22.5, 23.7)	0.99
Other multi-person hholds	37	23.7 (23.2, 24.2)	0.85	23 (22.3, 23.8)	0.99
One person < 60	86	23.6 (23.2, 24.1)	0.66	23 (22.6, 23.5)	0.99
One person aged 60 or over	135	23 (22.7, 23.4)	0.01	22.9 (22.5, 23.5)	0.99
No of persons in the household	–	–	**< 0.01** *	–	0.78
1	221	23.3 (23, 23.5)	–	23 (22.7, 23.4)	–
2	294	23.7 (23.5, 23.9)	0.03	23 (22.8, 23.3)	0.99
3	113	24.1 (23.5, 24.2)	0.01	22.7 (22.5, 23.2)	0.99
4	107	24.1 (23.8, 24.3)	**< 0.01**	23.2 (22.9, 23.4)	0.99
5	24	24.1 (22.8, 24.4)	0.33	22.6 (21.9, 23.7)	0.99
6 or higher	15	24.2 (23.5, 24.7)	0.12	23.4 (21.3, 24.1)	0.99
Age band of youngest person	–	–	**< 0.01**	–	0.17
0–4	71	24.1 (23.8, 24.3)	–	22.9 (22.5, 23.4)	–
5–10	68	24 (23.5, 24.3)	0.74	23.4 (22.5, 23.7)	0.86
11–15	50	23.9 (23.4, 24.3)	0.66	22.8 (22, 23.3)	0.77
16–24	73	24.2 (23.8, 24.6)	0.85	23 (22.6, 23.3)	0.95
25–59	233	23.7 (23.5, 24)	0.41	23 (22.8, 23.4)	0.86
60–74	213	23.4 (23, 23.5)	**< 0.01**	22.9 (22.6, 23.1)	0.95
75–84	54	23.5 (23.1, 24.2)	0.41	23.6 (23, 23.8)	0.25
85 or more	12	23.8 (22.6, 24.7)	0.66	22.9 (22, 24.1)	0.95
Age band of oldest person	–	–	**< 0.01**	–	**0.04**
16–34	53	24.4 (24, 24.8)	–	23.4 (22.9, 23.8)	–
35–49	202	24 (23.7, 24.2)	0.13	23 (22.6, 23.4)	0.17
50–59	159	23.5 (23.3, 23.8)	**0.01**	22.8 (22.6, 23.2)	0.17
60–74	259	23.4 (23.2, 23.7)	**< 0.01**	22.9 (22.6, 23.3)	0.21
75–84	83	23.6 (23.4, 24)	**0.02**	23.4 (23, 23.7)	0.90
85 or more	18	23.6 (22.8, 24.3)	0.11	22.9 (22, 24)	0.69

* Indicates groups where the assumption of equal variance was not met but stochastic dominance could be assessed. The bold font highlights cases where the *p* ≤ 0.05.

**Table 5.** Summary of the median standardised indoor temperatures (SIT), 95% confidence interval (CI) and significance test results. The *p*-values associated with each variable are the results the Kruskal–Wallis test and the *p*-values associated with each level of the variable are the result of the pairwise Mann–Whitney *U*-tests.

		Bedroom SIT (℃)		Living room SIT (℃)	
	*N*	Median	*p*-values	Median	*p*-values
**Employment status of HRP and partner**	–	–	**< 0.01**	–	0.12
One or more work full time	351	24 (23.7, 24.1)	–	23 (22.7, 23.2)	–
One or more work part time	65	23.9 (23.5, 24.3)	0.91	22.9 (22.5, 23.5)	0.65
None working, one or more retired	268	23.4 (23.2, 23.6)	**< 0.01**	23 (22.8, 23.2)	0.33
None working and none retired	90	23.7 (23.3, 24.2)	0.91	23.3 (22.8, 23.7)	0.08
Extended tenure of household	–	–	**0.02**	–	**< 0.01**
Own with mortgage	230	23.8 (23.5, 24.1)	–	22.7 (22.4, 23)	–
Own outright	270	23.5 (23.3, 23.7)	0.11	22.8 (22.6, 23)	0.31
Privately rent	62	24.1 (23.8, 24.6)	0.42	23.4 (22.9, 23.8)	**0.01**
Rent from LA	97	24 (23.6, 24.3)	0.70	23.7 (23.1, 23.9)	**< 0.01**
Rent from RSL	115	23.5 (23.3, 23.8)	0.26	23.5 (23, 23.7)	**< 0.01**
Anyone illness or disability	–	–	0.40	–	**0.02**
Yes	286	23.5 (23.5, 23.8)	–	23.1 (22.9, 23.5)	–
No	482	23.8 (23.6, 24)	0.28	22.9 (22.7, 23.1)	0.01
All households – income in 5 bands	–	–	**0.02**	–	**0.02**
Lowest 20%	156	23.5 (23.2, 23.8)	–	23.1 (22.9, 23.5)	–
Quintile 2	165	23.4 (23.3, 23.8)	0.98	23.1 (22.8, 23.5)	0.52
Quintile 3	162	23.7 (23.5, 24)	0.36	23 (22.7, 23.5)	0.53
Quintile 4	159	23.9 (23.6, 24.2)	0.05	23.1 (22.8, 23.4)	0.48
Highest 20%	132	24 (23.6, 24.2)	0.22	22.6 (22.3, 23)	0.01
Household vulnerable – on means tested or certain disability related benefits	–	–	0.47	–	**< 0.01**
Yes	250	23.6 (23.4, 23.8)	–	23.4 (23.1, 23.7)	–
No	524	23.8 (23.5, 23.9)	0.49	22.9 (22.7, 23)	**< 0.01**

**Table 6.** Summary of the median standardised indoor temperatures (SIT), 95% confidence interval (CI) and significance test results. The *p*-values associated with each variable are the results the Kruskal–Wallis test and the *p*-values associated with each level of the variable are the result of the pairwise Mann–Whitney *U*-tests.

		Bedroom SIT (℃)		Living room SIT (℃)	
	*N*	Median	*p*-Values	Median	*p*-Values
Dwelling type	–	–	**< 0.01** *	–	**< 0.01** *
Bungalow	97	22.8 (22.6, 23.1)	–	23.4 (22.9, 23.7)	–
Converted flat	15	22 (20.8, 23.2)	0.04	22.5 (21.7, 23)	**0.02**
Detached	137	23.8 (23.5, 24.1)	**< 0.01**	22.6 (22.3, 22.9)	**< 0.01**
End terrace	76	23.8 (23.5, 24.4)	**< 0.01**	23.1 (22.5, 23.6)	0.08
Mid terrace	121	24.1 (23.8, 24.3)	**< 0.01**	22.9 (22.5, 23.3)	**0.03**
Purpose built flat	101	23.7 (23.3, 24.2)	**< 0.01**	24 (23.6, 24.3)	**0.02**
Semi-detached	227	23.9 (23.7, 24.1)	**< 0.01**	22.8 (22.6, 23.1)	**< 0.01**
Dwelling age	–	–	**< 0.01**	–	**< 0.01**
Pre 1850	24	23 (21.6, 23.7)	–	21.2 (20.1, 22)	–
1850–1899	55	23.1 (22.3, 23.5)	0.85	21.8 (21.2, 22.5)	0.06
1900–1918	40	23.7 (23.1, 24.4)	**0.05**	22.5 (21.8, 22.9)	**< 0.01**
1919–1944	121	24 (23.8, 24.3)	**0.01**	23.3 (22.6, 23.5)	**< 0.01**
1945–1964	193	23.7 (23.4, 23.9)	**0.02**	23.1 (22.8, 23.3)	**< 0.01**
1965–1974	127	23.7 (23.5, 24.2)	**0.02**	23.4 (23, 23.6)	**< 0.01**
1975–1980	64	23.8 (23.4, 24.4)	**0.02**	23.7 (22.9, 24)	**< 0.01**
1981–1990	78	23.5 (23.3, 24.2)	**0.03**	23 (22.7, 23.6)	**< 0.01**
Post 1990	72	24 (23.6, 24.2)	**0.01**	23 (22.8, 23.6)	**< 0.01**
Floor area	–	–	**< 0.01**	–	**< 0.01** *
Less than 50 m^2^	80	23.5 (23, 24.2)	–	23.9 (23.5, 24.4)	–
50 to 69 m^2^	186	23.7 (23.5, 24.1)	0.58	23.4 (23, 23.6)	**< 0.01**
70 to 89 m^2^	202	23.9 (23.8, 24.2)	0.13	23.1 (22.8, 23.4)	**< 0.01**
90 to 109 m^2^	112	23.9 (23.4, 24.2)	0.26	23.1 (22.7, 23.4)	**< 0.01**
110 m^2^ or more	194	23.5 (23.2, 23.7)	0.58	22.4 (22.1, 22.5)	**< 0.01**
Storey	–	–	**< 0.01**	–	**< 0.01**
1st	97	22.8 (22.6, 23.1)	–	23.4 (22.9, 23.7)	–
2nd	577	24 (23.8, 24.1)	**< 0.01**	22.9 (22.8, 23.1)	**0.01**
3rd	73	23.3 (22.6, 23.8)	0.21	22.5 (21.9, 23.2)	**0.01**
4th	13	23.7 (23.1, 25.8)	**0.02**	23.8 (23.4, 26.2)	**0.05**
5th or higher	14	23.8 (21.5, 25.2)	0.18	24.5 (22.6, 25.5)	0.13
Traffic problems	–	–	0.36	–	0.29
No	728	23.7 (23.5, 23.8)	–	23 (22.9, 23.1)	–
Yes	46	24.1 (23.3, 24.4)	0.41	23.4 (22.4, 24)	0.31

* Indicates groups where the assumption of equal variance was not met but stochastic dominance could be assessed. The bold font highlights cases where the *p* ≤ 0.05.

**Table 7.** Summary of the median standardised indoor temperatures (SIT), 95% confidence interval (CI) and significance test results. The *p*-values associated with each variable are the results of the Kruskal–Wallis test and the *p*-values associated with each level of the variable are the result of the pairwise Mann–Whitney *U*-tests.

		Bedroom SIT (℃)		Living room SIT (℃)	
	*N*	Median	*p*-Values	Median	*p*-Values
Construction	–	–	0.38	–	**< 0.01** *
Solid masonry	156	23.8 (23.4, 24.1)	–	22.5 (22.1, 23)	–
Cavity masonry	532	23.7 (23.5, 23.8)	0.87	23 (22.9, 23.3)	**< 0.01**
Timber frame	30	24.1 (23.4, 24.8)	0.51	23.7 (22.8, 24)	**0.02**
Steel frame	13	23.1 (22.5, 24.9)	0.87	23.7 (22.6, 24.6)	**0.03**
Concrete frame	13	24.6 (21.2, 25.8)	0.51	24.6 (21.9, 26.8)	**0.01**
Concrete boxwall	17	23.7 (22.7, 24.4)	0.75	22.7 (21.4, 23.1)	0.89
Double glazing	–	–	0.06	–	**< 0.01**
No double glazing	47	23.5 (23.2, 24.3)	–	22.8 (22.1, 23.1)	–
Less than half	43	23.1 (22.8, 23.9)	0.45	22.3 (21.8, 22.7)	0.26
More than half	81	23.8 (23, 24.2)	0.73	23 (22.3, 23.6)	0.72
Entire house	603	23.8 (23.6, 23.9)	0.45	23.1 (22.9, 23.3)	0.26
Nature of area	–	–	**< 0.01**	–	**< 0.01**
City centre	16	24.2 (23.2, 24.9)	–	23.8 (21.9, 24.6)	–
Other urban centre	77	24.1 (23.7, 24.6)	0.94	23.5 (23, 23.8)	0.72
Suburban residential	515	23.8 (23.6, 24)	0.39	23 (22.9, 23.2)	0.21
Rural residential	102	23.4 (23.1, 23.8)	0.16	22.8 (22.5, 23.3)	0.21
Village centre	42	23.1 (22.8, 23.5)	**0.01**	22.6 (21.7, 23)	0.12
Rural	22	23.6 (21.9, 24.1)	0.16	22.5 (20.8, 23.5)	0.12
Main heating system	–	–	**< 0.01**	–	**< 0.01**
Boiler system with radiators	692	23.7 (23.5, 23.8)	–	23 (22.8, 23.1)	–
Storage radiators	53	23.5 (22.8, 24.1)	0.25	23.4 (22.6, 24)	0.11
Room heater	11	24.3 (22.5, 25.8)	0.31	23 (20.2, 26.3)	0.92
Communal	12	25.7 (24.4, 28)	**< 0.01**	25.8 (24.5, 27.5)	**< 0.01**
Loft insulation	–	–	0.07	–	0.65
None	20	24.2 (23.7, 24.7)	–	22.9 (21.8, 23.4)	–
Less than 100 mm	132	24 (23.7, 24.2)	0.80	23.3 (22.8, 23.6)	0.89
100 up to 150 mm	209	23.9 (23.7, 24.2)	0.67	23.1 (22.6, 23.4)	0.89
150 mm or more	337	23.5 (23.4, 23.8)	0.44	22.9 (22.8, 23)	0.89
SAP 09	–	–	0.50	–	**< 0.01** *
Less than 30	19	23.8 (22.5, 24.3)	–	22.5 (20.2, 23.3)	–
30 to 50	176	23.8 (23.5, 24.1)	0.60	23.3 (22.8, 23.5)	0.05
51 to 70	516	23.7 (23.5, 23.8)	0.60	22.9 (22.7, 23)	0.08
More than 70	63	24.1 (23.3, 24.4)	0.60	23.8 (23.4, 24.5)	**< 0.01**

* Indicates groups where the assumption of equal variance was not met but stochastic dominance could be assessed. The bold font highlights cases where the *p* ≤ 0.05.

**Table 8.** Summary of the *p*-values of the Fisher's exact test that tests the significance of association between categorical variables. A statistically significant association is assumed for *p* ≤ 0.05 and is indicated by the bold font.

	Age band of old persons	Age band of young persons	Income	Illness/ disability	Employment status	Tenure	Household. comp.	Vuln.	No. of persons
Construction	**0.04**	**0.03**	0.57	**0.05**	**0.04**	**< 0.01**	**0.02**	**< 0.01**	0.18
double glazing	0.60	0.82	0.37	0.71	**0.03**	**< 0.01**	0.89	**0.02**	0.61
Dwelling age	**0.05**	0.09	0.53	**0.02**	0.08	**< 0.01**	0.05	**0.01**	**0.04**
Dwelling type	**< 0.01**	**< 0.01**	**< 0.01**	**< 0.01**	**< 0.01**	**< 0.01**	**< 0.01**	**< 0.01**	**< 0.01**
Floor area	**< 0.01**	**< 0.01**	**< 0.01**	**0.03**	**< 0.01**	**< 0.01**	**< 0.01**	**< 0.01**	**< 0.01**
Loft insulation	**0.04**	**0.04**	0.26	0.45	**0.01**	**0.01**	0.24	**< 0.01**	**0.04**
Main heating system	**0.02**	**< 0.01**	**< 0.01**	0.10	**< 0.01**	**< 0.01**	**< 0.01**	**< 0.01**	**< 0.01**
Nature of area	0.53	0.92	0.12	0.68	0.09	**< 0.01**	**0.01**	**0.02**	0.08
SAP 09	0.20	0.19	**< 0.01**	0.45	0.27	**< 0.01**	0.06	**0.02**	**< 0.01**
Storey	**< 0.01**	**< 0.01**	**< 0.01**	**< 0.01**	**< 0.01**	**< 0.01**	**< 0.01**	**< 0.01**	**< 0.01**
Traffic problems	0.90	0.74	0.29	0.40	0.83	0.68	**0.01**	0.43	**< 0.01**

### Indoor overheating assessment

A total of 20 living rooms exceeded the threshold of Criterion 1, while 178 living rooms recorded some overheating hours. A similarly small number of dwellings failed Criterion 1 for the bedroom (19), while a greater number (284) experienced some hours of overheating. The extent of indoor overheating appears to be different when Criterion 2 is used, with 204 bedrooms having exceeded the static threshold. As part of the interviews conducted during EFUS, the occupants were asked whether they find it difficult to keep the bedroom cool. From a total number of 61 who responded positively, 29 were found to exceed the Criterion 2 threshold, 21 had some hours of overheating recorded while 11 had no hours recorded. The agreement between predicted and stated indoor overheating was lower when looking at Criterion 1 (the exact number is not provided to reduce the chance of identification).

### Household characteristics

Tables 4 and 5 summarise the median and 95% confidence intervals (CI) of the SIT for each household characteristic. The associated p-values indicate whether a statistically significant difference (if *p* ≤ 0.05) exists. For household composition, there was no significant difference in the living room, with the median SIT lying within a range of 0.2℃. On the contrary, the bedroom median SIT deviated significantly, with the value for a single occupant aged 60 or over being at 23℃ (CI (22.7, 23.4)℃), 1.1℃ lower than the maximum median bedroom SIT observed for this variable. The number of people in the household was significant only in relation to the bedroom SIT, with the median values generally increasing with the number of people. An association may exist between the SIT and the age band of the youngest and oldest occupant, as the older age bands experienced lower temperatures than the younger age bands. Households with no one working or one or more retiree had a lower median bedroom SIT than any other category under the employment status variable. A statistically significant effect was also observed for the household's tenure with regard to both the bedroom and living room SIT, with the living rooms of homes rented from a local authority being up to 1℃ warmer than homes owned with mortgage or outright (Figure 3). Households that might be considered vulnerable (by being on means tested or certain disability benefits) or where someone suffers from an illness or disability (but does not necessarily receive any benefits) had statistically higher median living room SIT. Statistically significant and opposite trends were observed for the income bands; the bedroom median SIT was slightly greater for higher income bands, while the opposite was true for the living room SIT.

### Dwelling characteristics

A summary of the median SIT and 95% CI for the dwelling characteristics along with the associated *p*-values is provided in Tables 6 and 7. Dwelling type and age, floor area, storey, construction and main heating system all appeared to have a statistically significant association with the SIT. Bungalows and converted flats had the lowest median SIT, while mid-terraced had the highest median bedroom SIT with 24.1℃ (CI (23.8, 24.3)℃) and purpose-built flats the highest median living room SIT with 24℃ (CI (23.6, 24.3)℃). Pre-1900 dwellings were overall cooler than post-1900 homes while the floor area of dwellings appeared to have a negative correlation with the median living room temperature. Storey was an important factor although the effect of increasing temperature with storey was greater for the living room than for the bedroom. The median SIT of dwellings with communal heating was 2℃ higher for the bedroom and 2.8℃ for the living room (Figure 3) compared to the more common gas boiler. Significant differences were also discovered for the terrain type, with urban dwellings being the warmest. The traffic problems variable was assumed to be a possible indication of local noise or air pollution that could deter occupants from keeping their windows open. However, it is also likely that dwellings whose occupants expressed traffic problems were located near the centre of the Urban Heat Island effect that is not captured by this analysis. Although a statistically significant result was not observed, occupants that were influenced by traffic problems had a median temperature 0.4℃ greater than the ones that did not experience traffic problems. A pattern of decreasing median bedroom SIT was observed with increased levels of loft insulation although no such pattern exists for the living room. The SIT was significantly different for different SAP ratings only in the living room; however, the median SIT was the highest in either room for a SAP rating > 70.

### Correlations between dwelling and household variables

Prior to any causation being attributed to individual variables analysed in Tables 4 to 7, any correlation between variables should be explored. Table 8 provides a matrix of *p*-values resulting from the Fisher's exact test with the null hypothesis of independent variables. By assessing the association of dwelling characteristics against household characteristics, a statistically significant association was obtained for each variable with at least one other variable. A further investigation in the suggested relationship between household vulnerability and floor area is displayed in Figure 4. With increased floor area, the percentage (and probability) of a dwelling's occupants being classified as vulnerable decreased.

## Discussion

Using the criteria defined within the methods section, approximately 2.5% of the 795 living rooms and 799 bedrooms failed Criterion 1, and 26% failed Criterion 2. These results were not in full agreement with the stated thermal discomfort of the occupants. Although the interviews did not necessarily take place during the summer and could, thus, be influenced by factors such as recall bias, the discrepancies between overheating criteria and stated thermal discomfort resemble previous findings.^32^ The large (175) number of dwellings that failed Criterion 2 while their occupants did not report thermal discomfort may provide further evidence of support to the ongoing discussion on the strictness of the 26℃ threshold.^25^ However, most dwellings that reported thermal discomfort in the bedroom did not exceed the threshold (32 of 61). As the summer of 2011 was relatively cool, with a mean summer (June–August) temperature across England of 14.8℃ (0.7℃ lower than the 1981–2010 average^47^) it could be hypothesised that a large percentage of English dwellings would fail Criterion 2 with the projected increase in summer temperatures.^3^

The analysis of household and dwelling characteristics generally confirmed the observations of previous studies^26,27,30^ and highlighted the differences between bedrooms and living rooms. As an example, increased levels of loft insulation appear to reduce indoor temperatures in bedrooms but have no clear effect on the living room. This is likely due to bedrooms being more frequently located directly under the roof and hence influenced more by the heat transfer through that surface. Thus, adding thermal insulation to a dwelling's loft may only reduce indoor overheating risk for the top-floor rooms. The living room SIT of dwellings rented from RSL or LA is significantly higher, in partial agreement with Hulme et al.^34^ A SAP rating > 70 was also associated with significantly higher living room SIT, resonating with the concerns of the unintended consequences of energy efficiency.^15,48^ The dwelling age, type, floor area and height were also statistically significant for both rooms in agreement with previous monitoring campaigns.^30,33^ The number of occupants and household composition had a strong influence on the bedroom temperatures but not on the living room. In agreement with Hulme et al.,^34^ simply the presence of young children and adults (indicated by the age band variables) was associated with greater bedroom temperatures. Households with occupants that are on means tested or other benefits also had statistically higher temperatures in the living room but lower in the bedroom. The choice of main heating system could be a key factor for indoor overheating risk, as dwellings with communal heating had a significantly greater indoor temperature in both rooms compared to dwellings with any other heating system. Although the sample size of dwellings with communal heating was small and the possibility of confounding variables exists, this result reinforces the importance of careful planning when designing and implementing communal heating systems.^49^

The multiple correlations between household and dwelling characteristics and the further investigation of the floor area and vulnerability association demonstrate the complexity of the indoor overheating problem. As the floor area increased, the living room SIT decreased. If considered independently, this might be expected since given the same solar and internal gains, a smaller room will reach a higher internal temperature. Another observation was that the median living room SIT of vulnerable occupants (on means tested or other disability benefits) was higher than that of non-vulnerable occupants. A plausible explanation is that individuals with disabilities may spend more time at home, resulting in increased internal gains and their limited mobility may lead to reduced ventilation.^27^ However, it was also observed that as the floor area decreased, there was an increased probability of an occupant being classified as vulnerable. If a reasonable explanation for the differences in temperature could be provided for either variable, which one is correct? It is expected that both factors and many more contribute to the observed differences and causation should be attributed with caution.

### Limitations

At the pre-processing stage, some data were eliminated on the base of faulty or misplaced loggers. However, it is possible that certain erroneous data remained within the analysed dataset. Furthermore, uncertainties may also arise from the answers provided during the interview and survey stage.

Although local weather data were used, they did not necessarily represent the ambient weather conditions at the exact location of each dwelling. This is especially true for dwellings located in urban areas, as the weather data may not effectively capture the influence of the urban heat island effect or the local microclimate.^50^

Performing the Kruskal–Wallis test at a significance level of 5% suggests that the null hypothesis may falsely be rejected (type I error) in 5% of the cases.^45,51^ Readers are thus advised to look at both statistical tests conducted, and the associated median SIT values provided for each variable.

The dataset analysed is the largest recent one that is currently available. Although weight factors for four variables (GOR, tenure, dwelling type and household working status) were provided to enable EFUS to be a broadly representative study of the English housing stock, the numerous other variables that could influence indoor temperatures limit the generalisability of this study's results. Finally, the correlation established between variables does not, of course, imply causation and conclusions should be drawn from this work with caution.

### Implications

The discrepancy between the occupants' stated thermal discomfort and the criteria-based overheating prediction may highlight the need to refine the thresholds for in-use studies within the industry. However, given the potential uncertainties resulting from the interview process, further evidence is required to support this action. As there are many factors that might influence the summer indoor temperatures, the use of dynamic building thermal simulations as suggested in TM59^20^ may indeed enable a better prediction of the indoor environment provided concerns regarding these tools and their inputs are addressed.^52,53^

Further academic work is required to address the complexity of the indoor overheating problem, especially for household and occupancy-related factors. Research on understanding the reasons behinds any significant results observed during this study, possibly through a mixture of detailed monitoring and further interviews will be greatly informative. In addition, refining the overheating criteria requires further work in defining domestic overheating in terms of temperature and potentially other variables.

In agreement with previous studies, the occurrence of indoor overheating in the existing housing stock even during a mild summer,^28^ in conjunction with the finding that indoor temperatures were highest for dwellings with a SAP rating > 70^34^ (possibly a consequence of increased fabric thermal insulation and airtighness^15,48^), reinforce the concerns regarding the current lack of indoor overheating assessment within the approved documents for the Building Regulations at the refurbishment stage and the limited guidance for new builds. With a warming climate, an ageing population and the subgroup of dwellings with the highest SAP rating being the warmest, the need to act is clear. Otherwise, alongside the risks to health, mechanical cooling is more likely to be widely adopted,^54^ increasing the summer energy demand and associated carbon emissions.

## Conclusions

The indoor overheating risk according to the criteria defined within Technical Memorandum 59 was estimated for the largest recent dataset currently available – the ‘*Energy Follow Up Survey*’. Although the prevalence of indoor overheating according to Criterion 1 was low, with only 2.5% of dwellings exceeding the threshold, almost 26% of dwellings failed Criterion 2, even during a relatively cool summer. Therefore, if these criteria were to be used for in-use assessments and with the projected increase in outdoor temperatures associated with climate change, a large percentage of dwellings will exceed the Criterion 2 threshold in the future.

By regressing the monitored indoor temperatures against the external weather conditions, the bedroom and living room temperatures were standardised to explore their correlation with the nine household and eleven dwelling characteristics. The bedroom standardised indoor temperatures were highest for the mid-terraced houses (24.1 (CI: 23.8, 24.3)℃) and the living room temperatures were highest for purpose-built flats (24 (CI: 23.6, 24.3)℃). The median living room temperature decreased with increased floor area, while the presence of children was associated with higher bedroom temperatures and so was the presence of occupants on means tested or certain disability benefits. Dwellings with SAP rating > 70 were the warmest, providing further support for the need of an indoor overheating assessment at the building design and refurbishment stage.

Importantly, multiple correlations between household and dwelling variables were also revealed. For example, with increased floor area, the likelihood of a dwelling's occupants being classified as vulnerable decreases. Therefore, drawing conclusions directly from individual variables should be approached with caution while further work is required to disentangle the complex relationships identified.
